# Left Atrial Posterior Wall Isolation with Pulsed Field Ablation in Persistent Atrial Fibrillation

**DOI:** 10.3390/jcm12196304

**Published:** 2023-09-29

**Authors:** Melanie A. Gunawardene, Gerrit Frommeyer, Christian Ellermann, Mario Jularic, Patrick Leitz, Jens Hartmann, Philipp Sebastian Lange, Omar Anwar, Benjamin Rath, Rahin Wahedi, Lars Eckardt, Stephan Willems

**Affiliations:** 1 Department of Cardiology and Intensive Care Medicine, Asklepios Hospital St. Georg, 20099 Hamburg, Germany; 2 Semmelweis University, 1082 Budapest, Hungary; 3 DZHK (German Center for Cardiovascular Research), Partner Site Hamburg/Kiel/Lübeck, 10178 Berlin, Germany; 4 Division of Electrophysiology, Department of Cardiovascular Medicine, University of Münster, 48149 Münster, Germany; christian.ellermann@ukmuenster.de (C.E.);

**Keywords:** pulsed field ablation, persistent atrial fibrillation, pulmonary vein isolation, left atrial posterior wall isolation

## Abstract

Background: Left atrial posterior wall isolation (LAPWI) may improve rhythm control in addition to pulmonary vein isolation (PVI) in persistent atrial fibrillation (persAF) patients undergoing catheter ablation (CA). However, LAPWI may be challenging when using thermal energy sources. Objective: This study aimed to investigate the efficacy and safety of LAPWI performed by non-thermal pulsed field ablation (PFA) in CA for persAF. Methods: Consecutive persAF patients from two German centers were prospectively enrolled. There were two study cohorts: (1) the LAPWI cohort, which included PFA-guided (re-)PVI with LAPWI for first-time and/or repeat ablation procedures; and (2) a comparative persAF cohort with a PFA PVI-only approach without LAPWI for first-time ablation within the same timeframe. Patients were followed up by routine Holter ECGs. Results: In total, 79 persistent AF patients were included in the study: 59/79 patients were enrolled in the LAPWI cohort, including 16/59 index (27%) and 43/59 repeat ablation procedures (73%). Sixteen patients (16/79; 21%) were in the PVI-only cohort without LAPWI. Of the patients treated with LAPWI, procedure time and fluoroscopy time was 91 ± 30 min and 15 ± 7 min, respectively. The acute PVI rate was 100% in all first-time ablation patients (32 patients (16 PVI only, 16 PVI plus LAPWI), 196/196 PVs). Of the 43 re-do patients in the LAPWI cohort, re-PVI was necessary in 33% (14/43) of patients (27 PVs; 1.9 PV per-patient); in 67% (29/43), all PVs were isolated, and antral ablation of the PV ostia was performed in 48% (14/29). LAPWI was performed successfully in all 59 (100%) patients of the LAPWI cohort. Two minor complications occurred. No esophageal lesion was detected in the LAPWI cohort (n = 33/59 (56%) patients underwent endoscopy). After 354 ± 197 days of follow-up, freedom from atrial arrhythmias was 79.3% (95-CI: 62–95%) in the complete LAPWI cohort (n = 14/59 (24%) on AAD: class Ic n = 9, class III n = 5). There was no difference regarding acute procedural and clinical outcome compared to the PVI-only cohort. Conclusion: LAPWI guided by PFA is feasible and safe in patients undergoing CA for persAF and shows favorable outcomes. In the context of durable PVI, PFA-guided LAPWI may be an effective adjunctive treatment option.

## 1. Introduction

Pulmonary vein isolation (PVI) is the cornerstone of index catheter ablation for both paroxysmal (PAF) and persistent atrial fibrillation (AF) [1]. Achieving sinus rhythm may be of prognostic relevance even in asymptomatic individuals with cardiovascular risk factors and recently diagnosed AF [2,3]. However, success rates of PVI in persistent AF have been reported to be as low as 54–59% in randomized trials [4,5]. Therefore, additional substrate modifications, including left atrial posterior wall isolation (LAPWI), that may improve rhythm control in persistent AF patients undergoing catheter ablation have been suggested. The left atrial posterior wall (LAPW) may play a role in AF initiation and maintenance as the pulmonary veins (PV) and LAPW share a common embryogenic origin and histology [6,7,8]. In addition, non-PV triggers and rotors from the LAPW have been described in previous studies [9,10,11].

Yet, up to now, adjunctive ablation strategies in addition to PVI, including linear ablation lines or substrate modification, even MRI-guided ablation, did not show any benefit over a PVI-only strategy in most randomized trials [4,5,12]. However, the recently published randomized ERASE-AF study indicated that individualized ablation of atrial low-voltage myocardium in combination with PVI significantly improved outcomes in persistent AF patients [13]. The randomized CONVERGE trial demonstrated higher rates of freedom from atrial tachyarrhythmias with an invasive hybrid epicardial and endocardial ablation approach (including PVI and LAPWI) compared to an endocardial-only ablation strategy (PVI, roof line and possible ablation of complex fractionated electrograms) in 153 patients with persistent and longstanding persistent AF [14].

Of note, achieving durable lesions with current thermal energy sources, such as RF, in patients undergoing endocardial catheter ablation may be difficult. The proximity to the esophagus needs to be considered when performing LAPWI for substrate modification [15]. Pulsed field ablation (PFA) has recently been introduced as non-thermal energy source for catheter ablation of AF with the advantage of myocardial tissue selectivity, theoretically sparing neighboring structures, such as the esophagus or phrenic nerve [16].

Performance of LAPWI with a recently introduced penta-spline PFA catheter was feasible and safe in a first-in-human study [17]. This penta-spline PFA catheter may offer an additional benefit when performing LAPWI due to the lesion size created by the large “footprint” of the device. Therefore, PFA could overcome two issues of thermal energy sources: (1) efficacy and (2) safety of LAPWI. Clinical data on this topic are sparse.

Thus, the objective of this prospective study was to investigate feasibility, safety and clinical outcomes of LAPWI performed with a penta-spline PFA catheter in a consecutive, real-world cohort of patients undergoing index or repeat ablation procedures for persistent AF in two high-volume EP centers.

## 2. Methods

### 2.1. Study Design

This study is a prospective study conducted in two German EP centers. Consecutive persistent AF patients, eligible for catheter ablation, were enrolled from May 2021 until March 2023.

The study was investigator-initiated without external funding; the authors are responsible for the design, execution and conduct of the study. The statistical analyses and interpretation of the data were approved by all authors, who attest to the accuracy of the data and the analyses. Written informed consent was obtained from all patients. The study was conducted in accordance with the provisions of the Declaration of Helsinki and its amendments. The institutional review board and ethics committee approved the study.

The study population consisted of two cohorts:

(1) The LAPWI cohort, including first-time and repeat ablation procedures in persistent AF patients. Ablation strategy included re-(PVI) with LAPWI performed by PFA.
(2) The comparative PVI-only cohort, including first-time ablation procedures in persistent AF patients. Ablation strategy included a PVI-only approach without LAPWI, performed by PFA.

### 2.2. Catheter Ablation—1. Procedural Workflow of LAPWI Cohort

**Figure 1:**
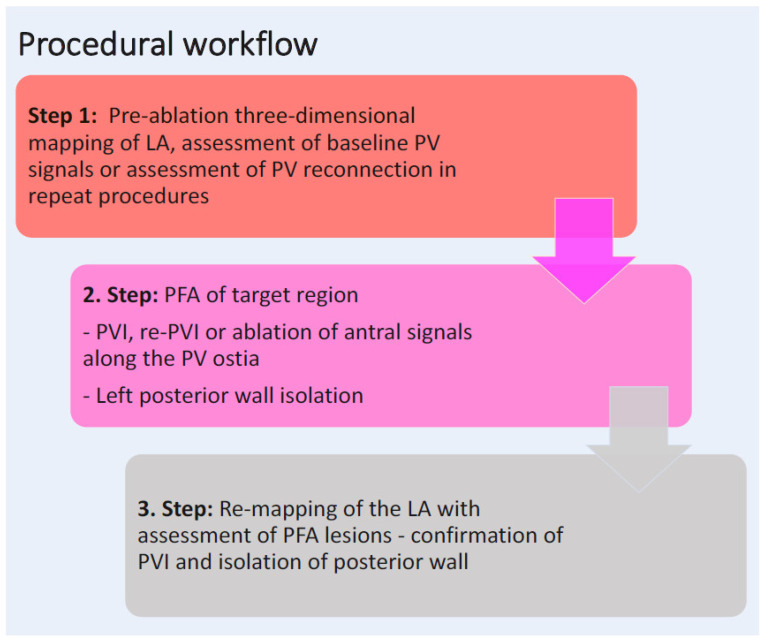
Procedural workflow in the LAPWI cohort. LA, left atrium; PFA, pulsed field ablation; PV, pulmonary vein; PVI, pulmonary vein isolation; RF, radiofrequency.

All persistent AF patients underwent PFA-guided catheter ablation with a 3D mapping system, either as an index or repeat ablation procedure (Figure 1) in the LAPWI cohort. Prior to ablation, intracardiac thrombus formation was ruled out by transesophageal echocardiography. Oral anticoagulation was given continuously or was minimally interrupted at the morning of the procedure and restarted 6 h after sheath removal as per the discretion of the responsible electrophysiologist. Procedures were performed under deep sedation using propofol and boluses of midazolam and sufentanyl or piritramid. Intravenous heparin (25,000 IU/5 mL) was applied to reach an activated clotting time of at least 300 s. To minimize vagal responses, 0.5–1 mg of intravenous atropine (0.5 mg/mL) was administered to all patients prior to PFA.

After femoral venous puncture (2 to 3 sheaths), single transseptal access was gained to the left atrium (LA), followed by pre-ablation three-dimensional mapping of the LA (Ensite X^TM^, Abbott, North Chicago, IL, USA or Rhythmia^TM^, Boston Scientific Corp., Marlborough, MA, USA). In index ablation procedures, PFA-guided pulmonary vein isolation (PVI), followed by left atrial posterior wall isolation (LAPWI), was conducted. LAPWI inherently included posterior roof ablation (therefore, rooflines are not separately described in this study).

In repeat ablation procedures, if required, PFA-guided re-PVI was performed. In case of isolated PVs, PFA was applied at the PV ostia to extend the antral ablation line and/or to ablate ostial signals in a subset of patients.

PFA was limited to LA ablation. If necessary, right atrial ablation, e.g., cavotricuspid isthmus (CTI) ablation, was conducted by RF current energy ablation.

Following PFA, 3D mapping of the LA was performed after ablation to assess acute ablation success and to confirm PVI and LAPWI (Ensite X, Abbott, North Chicago, IL, USA or Rhythmia^TM^, Boston Scientific Corp., Marlborough, MA, USA). Block of lines was assessed by post-ablation maps: sinus rhythm or paced rhythm was indicated by a block of conduction along the line, collision of wave fronts and/or by appropriate pacing maneuvers.

Esophagogastroduodenoscopy was performed post-procedurally in a subset of patients. Procedure-related complications were assessed. All patients received oral anticoagulation and treatment with proton pump inhibitors after ablation.

### 2.3. Catheter Ablation—2. Procedural Workflow of the Comparative PVI-Only Cohort

A comparative PVI-only cohort (explorative design) consisted of persistent AF patients undergoing PFA-guided PVI during the same timeframe. The before-mentioned workflow was the same, except no additional ablation was performed besides PVI with PFA. No LAPWI was performed in this cohort. Patients underwent first-time ablation; no repeat ablation procedures were included in this group. After PFA, entrance block of all PVs was assessed and confirmed.

### 2.4. Pulsed Field Ablation

In this study, ablation was performed by bipolar, biphasic PFA with a penta-spline catheter at 2.0 kV (Farawave^TM^, Boston Scientific, Marlborough, MA, USA). The PFA system, consisting of a generator, steerable sheath and an over-the-wire ablation catheter (FARAPULSE Pulsed Field Ablation system, Boston Scientific, Marlborough, MA, USA), has been reported in detail before [16,17]. Two different configurations of the same catheter—a *basket* and *flower* shape—are available. PVI or repeat PVI was performed using at least 8 applications at an output of 2.0 kV for all but 9 initial procedures, in which 1.9 kV was used (4 *basket*, 4 *flower* configuration). The catheter was slightly rotated between each pair of applications to ensure circumferential PV ostial and antral coverage [18]. If required, e.g., for anatomical constraints in catheter positioning, all 8 applications were performed in only one configuration (either *basket* or *flower*) [18]. LAPWI was conducted with the *flower* configuration and the guidewire retracted into the sheath. At least two applications were deployed at each site for LAPWI, with adequate overlap between catheter positions and PFA target sites [18]. During ablation, the PFA catheter was visualized in the electro-anatomical mapping system in a circular shape as reported before [18].

### 2.5. Follow-Up

Study participants underwent a structured follow-up including telephone calls, routine visits at the referring cardiologist and repetitive 24 h Holter ECG and/or rhythm monitoring via implanted cardiac devices, such as pacemakers and ICDs, if applicable.

### 2.6. Study Endpoints

The acute feasibility endpoint of the study was defined as successful catheter ablation with PFA, including acute isolation of PVs and LAPW, if applicable. Acute block of lines and acute isolation of LAPWI were assessed by post-ablation maps: sinus rhythm or paced rhythm indicated by a block of conduction along the line, collision of wave fronts and/or by appropriate pacing maneuvers, as well as the absence of any electrograms on the LAPW [18].

Secondary feasibility endpoints included freedom from AF, atrial flutter and/or atrial tachycardia after the 90-day blanking period. An atrial tachyarrhythmia recurrence was defined as AF/atrial flutter and/or atrial tachycardia recurrence of at least 30 s detected by any type of ECG or implantable cardiac device.

The acute safety endpoint included all periprocedural major and minor adverse events during hospitalization.

### 2.7. Statistics

Continuous data are shown as median and interquartile range or as mean ± standard deviation. Student’s *t*-test (paired or unpaired) or the Mann–Whitney U test for unpaired variables were used for group comparisons. Categorical data are expressed as absolute and relative frequencies; they were compared with the chi-squared or Fisher’s exact test. Statistical significance was assumed at a *p*-value < 0.05. Statistical analyses were performed with the GraphPad Prism 9.0 software (GraphPad Software Inc., San Diego, CA, USA).

## 3. Results

In total, 79 persistent AF patients were included in the study: 59 (79%) in the LAPWI cohort and 16 (21%) in the PVI-only cohort without LAPWI. The results are presented by each cohort.

### 3.1. LAPWI Cohort—Baseline Characteristics

A total of 59 persistent AF patients were enrolled in the LAPWI cohort, including 16 index (27%) and 43 repeat ablation procedures (73%). Baseline characteristics of the LAPWI cohort are shown in Table 1. Re-do patients underwent a median of two [q1–q3: 2–3] prior ablation procedures with a maximum of seven prior ablations in one patient. The mean age was 64 ± 14 years, and 78% (46/59) of patients were male. Of the 43 patients undergoing repeat ablation with PFA in this study, prior ablation procedures consisted of 2.3 ± 0.8 PVI and/or re-PVI attempts per patient with RF and/or cryo-energy. Prior RF lesion sets included anterior line ablation in 7/43 (16%), roof line in 5/43 (12%), mitral isthmus line in 2/43 (5%), posterior wall ablation in 4/43 (9%), ablation of complex fractionated electrograms in 11/43 (26%) and right atrial isthmus ablation in 21/43 (49%) patients.

### 3.2. LAPWI Cohort—PFA Ablation Procedure

**Figure 2:**
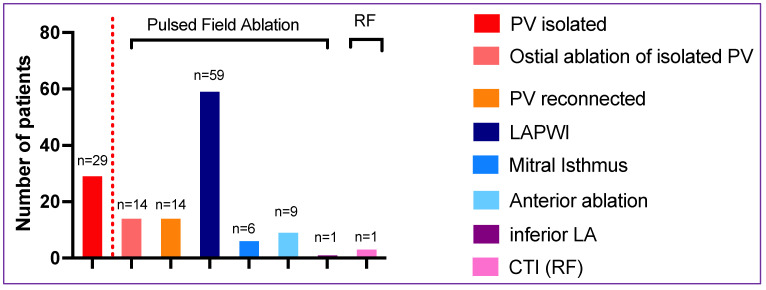
Ablation strategies. The number of patients with isolated PVs at repeat ablation are shown on the left side of the graph. On the ride side of the dotted line, ablation strategies of the complete cohort (n = 59) are shown. CTI, cavotricuspid isthmus ablation; LA, left atrial; LAPWI, left atrial posterior wall isolation; PV, pulmonary vein.

In the LAPWI cohort, skin-to-skin procedure time and fluoroscopy time were 91 ± 30 min and 15 ± 7 min, respectively. Left atrial dwell time for the PFA catheter was 44 ± 17 min with a LA fluoroscopy time of 9 ± 5 min. Three-dimensional maps were created in all procedures (59/59, 100%), including high-density mapping of the LA in 66% (39/59) of patients. Procedural details of the LAPWI cohort are shown in Table 2. De novo PVI for index procedures was performed in 16 patients with a 100% acute PVI rate (68/68 PV, including 4 right middle PVs) with a median of 32 (q1–q3: 32–35) PFA applications for all PVs per patient (Figure 2). In patients undergoing a repeat ablation procedure, PVI durability was 67% (29/43) on a per-patient basis. PV reconnections warranting re-isolation were found in 27 PVs of 14 patients (1.9 PV per patient); this included the LSPV in 7/14 (50%), LIPV in 4/14 (29%), RSPV in 6/14 (43%) and RIPV in 10/14 (71%) patients. An average of 9 ± 2 PFA applications were performed to re-isolate these PVs. Of the 29 patients presenting with all PVs durably isolated, 14/29 (48%) received PFA applied to the PV antra to extend the level of isolation proximally and/or to ablate antral signals.

**Figure 3:**
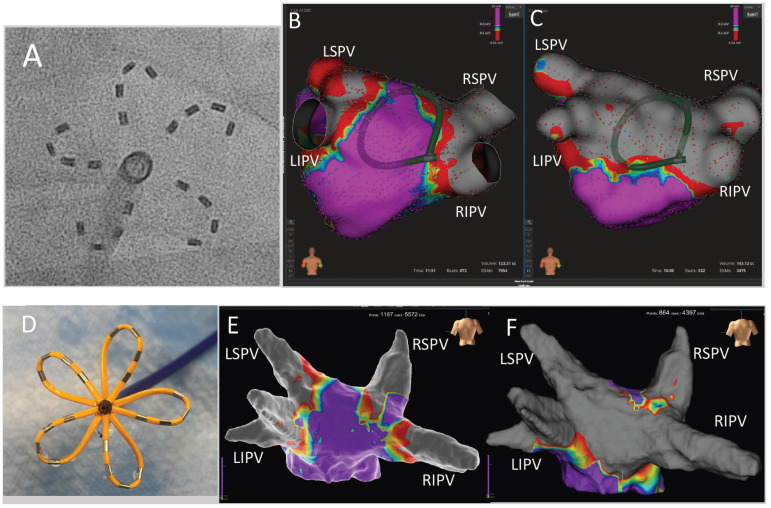
Left atrial posterior wall isolation with pulsed field ablation in patients with isolated pulmonary veins. (**A**) Fluoroscopic image of the PFA catheter in flower configuration (guidewire retracted for LAPWI). (**B**) High-density 3D map of the left atrium from a posterior view in a patient with isolated PVs before PFA. The PFA catheter is positioned at the ablation site in flower configuration and visualized as a circular shape. (**C**) High-density 3D map of the left atrium from the same patient after successful PFA-guided LAPWI (voltage maps cut-off: 0.1–0.3 mV). (**D**) Photographic image of the penta-spline PFA catheter in *flower* configuration (guidewire retracted for LAPWI). (**E**) High-density 3D map of the left atrium from a posterior view in a different patient with isolated PVs before PFA. (**F**) High-density 3D map of the left atrium from the same patient after successful PFA-guided LAPWI (voltage maps cut-off: 0.1–0.5 mV). LIPV, left inferior pulmonary vein; LSPV, left superior pulmonary vein; RSPV, right superior pulmonary vein; RIPV, right inferior pulmonary vein.

**Figure 4:**
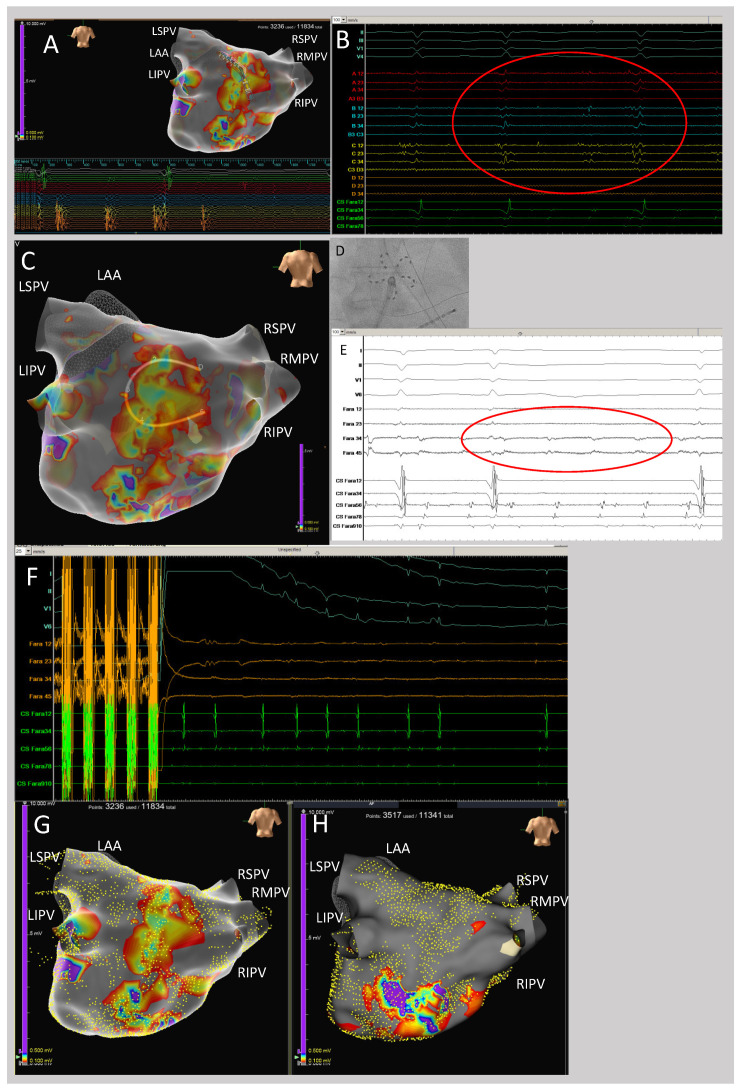
Atrial fibrillation termination during left atrial posterior wall isolation. (**A**) High-density 3D map of the left atrium from a posterior view during atrial fibrillation. Note the extensive scarring of the left atrial posterior wall with fractionated electrograms depicted by the mapping catheter (**A**,**B**). (**B**) Example of fractionated electrograms depicted by the multipolar mapping catheter at the left atrial posterior wall. (**C**) Three-dimensional high-density map of the left atrium from a posterior view during atrial fibrillation with the PFA catheter positioned at the posterior wall in the flower shape prior to ablation. The penta-spline PFA catheter is visualized in a circular shape via impedance tracking. (**D**) Fluoroscopy image of the PFA catheter in flower shape positioned at the left atrial posterior wall. (**E**) Example of fractionated electrograms depicted by electrodes of the PFA catheter at the left atrial posterior wall. (**F**) Shortly after PFA at the left atrial posterior wall, atrial fibrillation terminated to sinus rhythm. Of note, cardioversion of AF was not possible before. (**G**) Pre-ablation high-density map of the left atrial posterior wall (yellow points indicate the mapped surface points). (**H**) Post-pulsed field ablation high-density map demonstrating successful left atrial posterior wall isolation with homogenization of the posterior scar and elimination of the fractionated electrograms (yellow points indicate the mapped surface points). For all maps, the voltage cut-off was 0.1–0.5 mV. LAA, left atrial appendage; LIPV, left inferior pulmonary vein; LSPV, left superior pulmonary vein; RSPV, right superior pulmonary vein; RIPV, right inferior pulmonary vein.

Successful LAPWI with PFA was performed in all patients of the LAPWI cohort (59/59; 100%), with a median of 19 (q1–q3: 10–26) PFA applications in the *flower* configuration per patient (Figure 3 and Figure 4). There were no acute reconnections of LAPWI during the study procedures.

Limited additional left atrial substrate modification with PFA was conducted in 12/59 (20%) patients in low-voltage areas indicating left atrial scarring (performed in repeat ablation procedures only), including anterior line (n = 9), mitral isthmus line (n = 6) and infero-septal LA (n = 1) with a median of 10 (q1–q3: 7–18), 8 (q1–q3: 4–11) and 10 PFA applications, respectively (Figure 2). Due to extensive prior scarring, one consecutive isolation of the left atrial appendage occurred during PFA (1/59; 17%). Three patients (3/59; 5%) received additional CTI ablation with RF.

### 3.3. LAPWI Cohort—Procedural Adverse Events

No major complication occurred in the LAPWI cohort. Two minor complications were reported: one small groin arteriovenous fistula, not requiring treatment, and one transient coronary spasm during ablation at the mitral isthmus that was resolved with intracoronary application of nitroglycerin (Table 2). No phrenic nerve palsy or vagal response was reported. Thirty-three patients (33/59, 56%) underwent post-procedural endoscopy one day after PFA; no esophageal lesion was detected after LAPWI in any patient.

### 3.4. Results—Comparative PVI-Only Cohort

A total of 16 persistent AF patients underwent an index PVI-only approach with PFA without LAPWI during the same time frame. All procedures were index AF procedures (16/16, 100%), with no prior ablation was performed. Baseline characteristics and procedural data of the PVI only cohort are shown in Table 3. Compared to the LAPWI group, patients of the PVI-only cohort were less often on antiarrhythmic drug therapy (n = 3/16 vs. 31/59 patients, *p* = 0.0227) *p* = 0.18) prior to ablation and showed a non-significant trend in a shorter AF history (median 2 [0.5–6] vs. 8 [3–136], p = 0.18). Procedure times were non-significantly shorter (76 ± 31 vs. 91 ± 30 min, *p* = 0.095) in the PVI-only cohort, as shown in Table 3.

Of the comparative PVI-only cohort, six patients (38%, 6/16) were re-hospitalized during follow-up (including early recurrences in the blanking period; of the 6/16, one patient was re-hospitalized due to renal failure, and one patient is scheduled for repeat ablation). One patient was on flecainide due to arrhythmia recurrence.

### 3.5. Follow-Up—Complete Study Cohort

Fifty-eight (58/59, 98%) patients of the LAPWI cohort and all sixteen patients (100%) of the PFA PVI-only cohort passed the 90-day blanking period and were included in the following analysis. Mean follow-up time was 354 ± 197 days.

**Figure 5:**
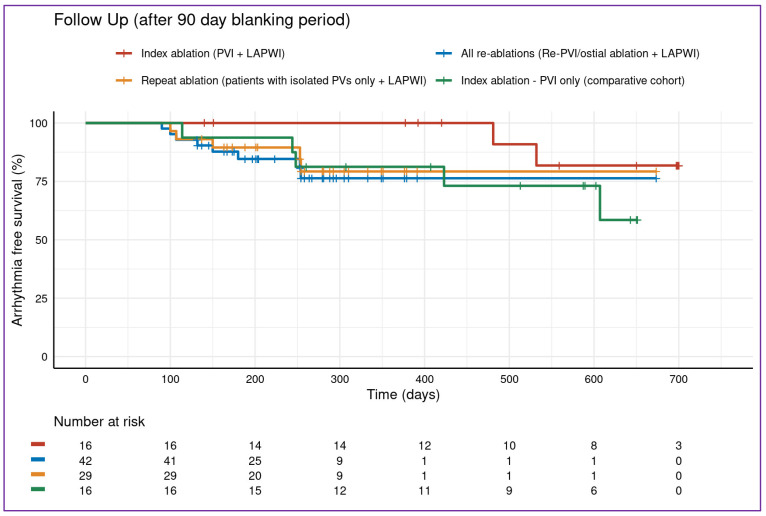
Follow-up. Follow-up of the 43/59 (73%) patients of the study cohort that passed the 90-day blanking period and were included in the follow-up analysis. There was no difference between the groups (*p* = 0.367 between index and repeat ablation procedures). LAPWI, left atrial posterior wall isolation; persAF, persistent atrial fibrillation; PVI, pulmonary vein isolation.

Regarding the LAPWI cohort, Kaplan estimates for freedom from atrial arrhythmias were 76.9% (95%-confidence interval (CI) [62–95%]) for all LAPWI patients at 500-day follow-up (index and repeat ablation, Figure 5). Within the LAPWI cohort, Kaplan estimates were 76.3% (CI [63–93%]) for repeat ablation patients with LAPWI, 79.3% (95%-CI [64–98%]) for patients with durably isolated PVs plus LAPWI and 82% (95%-CI [62–100%]) for patients with index PVI plus LAPWI.

For patients of the PVI-only cohort not receiving LAPWI, the Kaplan–Meier estimate for freedom from atrial arrhythmia was 73% (95%-CI [54–100%]).

There was no difference in freedom from atrial arrhythmias between index and repeat ablation procedures (*p* = 0.367).

Antiarrhythmic drug (AAD) use was present in 20% of patients of the complete study cohort (n = 15/75), (Figure 5).

There were 10 symptomatic arrhythmia recurrences (8/10 AF, 2/10 atrial tachycardia) in the LAPWI cohort (10/59; 17%: with 2/16 (13%) index PVI plus LAPWI ablation patients and 8/58 (14%) redo patients). Five of the patients from the comparative index PVI-only cohort presented with atrial arrhythmia recurrence during follow-up (5/16; 31%).

Of all LAPWI patients, 24% (14/59) were still on antiarrhythmic drugs (AAD) after the 90-day blanking period (class Ic n = 9, class III n = 5), with 2 of the 14 patients suffering from atrial tachyarrhythmia recurrence. Of all sixteen index PVI plus LAPWI ablation patients, only one (1/16; 6.25%) was on AAD (amiodarone) because of arrhythmia recurrences. Thus, 15/16 index PVI plus LAPWI persistent AF patients were off AAD during follow-up after the blanking period.

Of the 43 patients undergoing re-ablation, 13 (30%) were still on AAD (class Ic n = 9, class III n = 4) after the blanking period. Of these 13 patients, only one (1/13; 7.8%) had atrial tachyarrhythmia recurrence and was treated with propafenone. Of the 29 patients with durable PVI during repeat ablation, all passed the 90-day blanking period and showed 83% (24/29) freedom from atrial arrhythmia (Figure 5).

During follow-up, 76% (45/59) of patients in the LAPWI cohort remained completely asymptomatic. Fourteen LAPWI patients (24%, 14/59) were re-hospitalized for electrical cardioversion (including early recurrences in the blanking period (12/59) or another catheter ablation procedure (2/59)) during follow-up.

## 4. Discussion

### 4.1. Major Findings

The major findings of this study are as follows:

LAPWI in persistent AF is feasible in index and repeat ablation procedures using a large “footprint”, penta-spline PFA-catheter in a prospective real-world cohort.

LAPWI is safe, especially in regard to potential esophageal lesions.

The follow-up of this study shows favorable outcomes.

In a subset of patients with durable PVI, LAPWI showed high rates of freedom from atrial tachyarrhythmias offering a possible treatment option for persistent AF patients in this setting.

### 4.2. Left Atrial Posterior Wall Isolation with PFA

As shown in our study, procedural performance of LAPWI using a large “footprint”, penta-spline PFA-catheter is feasible, fast, comparably simple and safe. In our prospective real-world cohort, 100% of LAPWs were acutely isolated within a procedure time of 91 ± 30 min (including two centers and several operators), compared to much longer procedure durations of 142 ± 69 min in the CAPLA study investigating LAPWI performed by radiofrequency (RF) current energy [12]. Regarding PFA efficacy, Reddy et al. [17] demonstrated not only 100% acute (25/25 patients) but also 100% chronic (21/21 patients) LAPWI—via prospective 3-month remap procedures—in the *Persafone* clinical feasibility study [17]. Median ablation time to achieve PFA-guided LAPWI in this study was fast, requiring only 10 min (q1–q3 6 to 13 min) [17]. These results emphasize the simplicity of a PFA-guided approach to isolate the LAPW.

Even in the largest published clinical data set investigating PFA to date—the MANIFEST-PF registry—45.8% of European electrophysiologists performed PFA-guided LAPWI “sometimes” during their procedures [19]. Therefore, LAPWI seems to have found a way into clinical routine in Europe. The findings of our study support this rationale with evidence of feasibility and safety in a real-world cohort.

### 4.3. Thermal Energy Sources for LAPWI

As mentioned in the introduction, most previous randomized trials showed no benefit of addressing the atrial substrate outside the pulmonary veins in patients undergoing catheter ablation for persistent AF [5,13]. However, due to the limited success of PVI in persistent AF, additional ablation strategies are needed. Although most studies included in a pooled analysis favored additional LAPWI performed by thermal energy sources [20], conflicting evidence from trials and meta-analyses exists regarding whether it is a beneficial ablation strategy [21].

For example, the randomized CAPLA study found no benefit of RF-guided PVI plus LAPWI compared to PVI alone, with freedom from recurrent atrial arrhythmias of 52.4% vs. 53.6% (hazard ratio 0.99 [95%CI, 0.73–1.36]; *p* = 0.98) at 12 months, respectively [12]. Of note, in this initially persistent AF cohort, the majority of AF recurrences were of paroxysmal character (62.8% PVI plus LAPWI group and 61.5% PVI-alone group), and the median AF burden at 12 months was 0% in both groups, proving catheter ablation itself (with and without LAPWI) to be an overall effective rhythm control strategy in persistent AF patients [12].

Although additional LAPWI showed no effect on patients’ outcome, some issues need to be addressed and may explain the negative outcome of the CAPLA trial. Acute ablation success of LAPWI was only 86.5%, including focal ablation within the box lesion, e.g., to target epicardial connections [12]. A major limitation leading to the failure of LAPWI in 23 patients was a significant rise in the esophageal temperature to a median of 39.3 °C (IQR, 38.8–39.9 °C) [12], resulting in incomplete RF lesions. Additionally, of the 16 patients (9.4%) in the PVI plus LAPWI group receiving repeat ablation, 69% (11/16) demonstrated reconnection of the LAPW. In a study by Worck et al., patients underwent systematical remapping at 6 months after RF-guided LAPWI, demonstrating that more than half of the patients (54%) showed reconnection of the posterior wall [22]. Even the attempt to turn up the power in the PEF-HOT study and randomizing patients to PVI and LAPWI or PVI alone with a high-power short-duration RF protocol did not show any benefit of additional LAPWI. The study was terminated early due to futility [23].

From this perspective, the assessment of a benefit from LAPWI is strongly limited by being unable to effectively isolate the LAPW with thermal energy ablation.

This obstacle of limited ablation success on the posterior wall could be overcome easily with PFA, allowing for more extensive ablation due to its tissue electroporation threshold [24].

PVI in combination with LAPWI was associated with a numerical lower rate of arrhythmia recurrence after first-time ablation compared to a PFA PVI-only approach (13% (2/16) versus 31% (5/16) recurrences) in our study. Limited by the small explorative sample size in our study, more data on PFA-guided LAPWI and randomized trials are therefore needed to assess the true benefit of additional and efficient LAPWI.

### 4.4. Safety Regarding the Esophagus

Although severe injuries of the esophagus causing major complications after catheter ablation are rare, esophageal injuries are still one of the main safety issues to be considered when performing AF ablation with thermal energy sources. Endoscopically detected esophageal lesions have been described in up to 30% of cases after catheter ablation [25]. Additionally, mediastinal edematous alterations have been detected in 70% by endoscopic ultrasound following cryoballoon ablation [26].

Especially, when addressing the LAPWI as an ablation target, concerns of causing damage to the esophagus arise due to the proximity of these two structures to each other. Although LAPWI with RF has been reported to be safe [12,23,27], the potential risk of harming the esophagus cannot be eliminated. In the randomized CAPLA study, no esophageal complication was reported. In a meta-analysis including 807 LAPWI patients, adverse events were not different to a PVI-only approach [28]. In a study by Worck et al., two esophageal lesions were found during a post-procedural endoscopy of 24 patients after LAPWI performed by RF in a persistent AF cohort [22].

In our study, no esophageal lesion was found during post-procedural endoscopy after LAPWI. Up to now, world-wide, there has been no published report of an esophageal lesion following ablation with this study’s PFA system. Therefore, PFA may show a favorable safety profile, sparing the esophagus.

### 4.5. PFA in the Setting of Re-Ablation

Effective ablation with RF can be challenging in patients undergoing repeat ablation procedures due to the presence of heterogeneous scarring that facilitates conduction gaps [29]. In a preclinical study, RF ablation in scarred myocardium resulted in limited and unpredictable effects, likely related to the heterogeneous effects of thermal injury in this complex substrate (mix of healthy, scarred and pre-ablated myocardium) [29].

Additionally, RF ablation on scarred ventricular myocardium showed less uniform lesions that were largely limited to the subendocardium compared to transmural, uniform and well-demarcated lesions exhibiting irreversible injury by PFA [30]. Another preclinical study showed that PFA on top of a chronic PFA lesion (4 weeks earlier) was able to create equally deep lesions compared to PFA on healthy myocardium [31]. Another recently published study of Kawamura et al. was able to show that PFA penetrated both infarct and iatrogenic scars (after RF ablation) successfully to create deep lesions in an animal model [32]. In our study, PFA in repeat ablation procedures after prior thermal ablation showed favorable rates of freedom from arrhythmia with 76.9% in persistent AF patients. Therefore, a switch from thermal energy sources to non-thermal PFA could be of benefit in regard to lesion formation and procedural effectiveness in patients undergoing repeat ablation for AF recurrence. However, more pre-clinical and clinical studies are needed to investigate this hypothesis.

### 4.6. Durable Pulmonary Vein Isolation

Furthermore, in the setting of isolated pulmonary veins, strategies to ablate persistent AF are warranted. In the era of increasing effectiveness, this population will be of emerging relevance. Effective LAPWI could play a role in these patients [7]. In our study, PVI durability was found in 67% of patients undergoing repeat catheter ablation, demanding additional substrate modification. PFA-guided LAPWI was performed effectively and safely in this patient cohort, showing a favorable outcome.

Moreover, in patients undergoing repeat ablation after initial PFA-guided PVI, a 91% PVI durability rate has been reported in a real-world, single-center experience, inclusive of the learning curve [33]. Therefore, PV reconnections could become less of a concern in patients with recurrent atrial arrhythmias after initial PFA. Tohoku et al. showed that after PFA PVI, a majority of patients receiving repeat ablation presented with a macro re-entry tachycardia [33]. The critical isthmus was found to be linked to the initial lesion set at the LAPW in half of the AT mechanisms [33]. Additionally, 72.7 ± 19.0% of the area of the LAPW was shown to be ablated after initial PFA with the penta-spline PFA catheter [33]. Unintentional ablation of the LAPW with the penta-spline PFA catheter may be explained by the catheter design [18]: due to the mechanical features of the over-the-wire PFA catheter, the catheter tends to turn posteriorly and to the roof when advanced to the PV ostium, away from the anterior aspect of the PV antrum [18]. Therefore, a maneuver in which the sheath is rotated actively towards the anterior aspect of the PV ostium to avoid unintentional ablation of the LAPW may be considered.

One could argue that additional ablation of the LAPW in persistent AF patients undergoing initial PFA-guided PVI could avoid such posterior wall-dependent AT recurrences. However, electrophysiologists may implement strategies atop the usual learning curve development, e.g., future 3D integration of the catheter or visualization by intracardiac echocardiography, to avoid unintended ablation of the LAPW.

### 4.7. Future Directions

Treatment of persistent AF with LAPWI performed by PFA showed high acute and chronic success in our explorative, prospective study.

Yet, PFA is still a novel ablation technology first introduced to the European market in 2021. Currently, the results of the ADVENT trial, comparing PFA to thermal energy sources (RF and cryoballoon ablation), showed non-inferiority of PFA for paroxysmal AF patients regarding a composite endpoint, including freedom from atrial arrhythmias [34]. Following this, the ongoing ADVANTAGE AF Study (NCT05443594) intends to establish the safety and effectiveness of PFA for the treatment of drug-resistant, symptomatic persistent AF.

Beyond PFA-guided PVI, larger multicenter trials are needed to assess the feasibility and safety of PFA-guided LAPWI as a strategy to treat persistent AF in first-time and repeat ablation procedures systematically. Additionally, comparisons of PFA-guided LAPWI to alternative strategies for persistent AF treatment are lacking and need to be investigated in a randomized fashion.

### 4.8. Limitations

The present study yields some limitations. The design of our two-center study was explorative, and therefore, the sample size in the current study is small, limiting the impact of the results. Also, the follow-up of this study did not include continuous or daily ECG monitoring, which might lead to the missing of short paroxysmal AF episodes. Another limitation is that no quality-of-life questionnaires were provided during follow-up and patients were only asked about symptom improvement after ablation in a dichotomous assessment. Further, in regard to healthcare utilization, only re-hospitalization data were available, while no data about healthcare costs were available.

We present only acute remapping data on acute LAPWI. There is no chronic remapping information compared to previously published studies about LAPWI [17], and therefore, there is no information regarding chronic PFA lesion patterns in our study.

The study is not randomized and there is no control group, including thermal energy sources. However, we provided comparative data of a PFA-guided PVI-only approach in persistent AF patients that underwent first-time ablation.

The study includes the learning curve of the novel penta-spline PFA system and, owing to the parameter-specific nature of PFA, the results described herein cannot be conflated with other PFA systems.

## 5. Conclusions

LAPWI guided by PFA is fast, feasible and safe—most notably for the esophagus—in patients undergoing first and repeat ablation for persistent atrial fibrillation and shows favorable clinical outcomes. Especially in patients with durable pulmonary vein isolation, PFA-based LAPWI could be an effective adjunctive treatment option. In the field of catheter ablation for persistent atrial fibrillation, PFA-guided LAPWI can therefore overcome considerable obstacles of thermal energy sources regarding both safety and efficacy.

## Figures and Tables

**Table 1:** Baseline Patient Characteristics of LAPWI-treated patients.

	LAPWI n = 59
Age (years)	64 ± 14
Male gender, n (%)	46 (78)
Hypertension, n (%)	37 (63)
BMI (kg/m^2^)	28 ± 4
CHA_2_DS_2_-VASc score	2 [1–4]
Persistent AF, n (%)	59 (100)
History of AF (years)	8 (3–13)
Prior AAD, n (%)	31
- Class Ic, n (%)	20
- Class III, n (%)	11
Prior catheter ablation procedures (n = 43) *	2 (2–3) [max. 7]
Left atrial diameter (mm)	45 (42–50)
Left ventricular function, n (%)	
- Normal (ejection fraction 50–70%) - Mild dysfunction (40–49%) - Moderate dysfunction (30–39%) - Severe dysfunction (<30%)	44 (75) 5 (8) 6 (10) 4 (7)

Values are mean ± standard deviation, median [first-third quartile] or n (%). * for patients undergoing repeat ablation only. AAD, antiarrhythmic drug; AF, atrial fibrillation; BMI, body mass index. CHA_2_DS_2_-VASc score is a clinical estimation of the risk of stroke in patients with atrial fibrillation; scores range from 0 to 9, with higher scores indicating a greater risk of stroke = congestive heart failure, hypertension, age > 75 years, diabetes, previous stroke, transient ischemic attack, or thromboembolism, vascular disease, age 65–75 years, and sex category. PAF, paroxysmal atrial fibrillation.

**Table 2:** Procedural data and periprocedural complications of LAPWI-treated patients.

	All n = 59
**Procedural Data**	
Initial Rhythm	
Sinus rhythm, n (%)	29 (49)
Atrial fibrillation, n (%)	29 (49)
Atrial tachycardia, n (%)	1 (2) *
Electrical cardioversion prior to ablation (only in the patients presenting in AF/AT, n (%)	23 (77)
Total procedure time (min)	91 ± 30
Total fluoroscopy time (min)	14 ± 7
Three-dimensional mapping, n (%) High-density mapping, n (%)	59 (100) 39 (66)
Patients with visualization of PFA catheter in mapping system, n (%)	59 (100)
**Pulsed Field ablation**	
Time of PFA catheter in the left atrium (min)	44 ± 17
Fluoroscopy time during PFA (min)	9 ± 5
**Total PFA applications per PV per patient**	
**LSPV**	8 [8–8]
**LIPV**	8 [8–8]
**RSPV**	8 [8–8]
**RIPV**	8 [8–8]
**Additional PFA applications**	
LAPWI (n = 59)	19 [10–26]
Mitral isthmus line (n = 6) Anterior line (n = 9) Inferior LA (n = 1)	8 [4–11] 10 [7–18] 10
**PFA catheter size **	
31 mm 35 mm	50 (85) 9 (15)
**Additional RF ablation **	
CTI ablation	3 (5)
**All complications, n (%)**	2 (3.4)
Pericardial tamponade, n (%)	0 (0.0)
Stroke/TIA	0 (0.0)
Phrenic nerve palsy, n (%)	0 (0.0)
Esophageal lesion, n (%)	0 (0.0)
Coronary spasm, n (%)	1 (0.0)
Minor groin complication (small AV fistula), n (%)	1 (0.0)

Values are mean ± standard deviation, median [first-third quartile] or n (%). * patient presented with atrial tachycardia; during entrainment the atrial tachycardia degenerated to atrial fibrillation. A, atrial fibrillation; AT, atrial tachycardia; LA, left atrium/atrial; LAPWI, left atrial posterior wall isolation; LIPV, left inferior pulmonary vein; LSPV, left superior pulmonary vein; min, minutes; PFA, pulsed field ablation, PVI, pulmonary vein isolation; RSPV, right superior pulmonary vein; RIPV, right inferior pulmonary vein.

**Table 3:** Baseline Patient Characteristics and Procedural Data of PVI-only cohort.

Baseline Data	PVI Only n = 16	Comparison to LAPWI Cohort *p*-Value
Age (years)	69 ± 9	0.2
Male gender, n (%)	10 (63)	0.2
Hypertension, n (%)	12 (75)	0.6
BMI (kg/m^2^)	26 ± 5	0.4
CHA_2_DS_2_-VASc score	2 [1–4]	0.5
Persistent AF, n (%)	16 (100)	0.1
History of AF (years)	2 [0.5–6]	0.18
Prior AAD, n (%)	3 (19)	0.02
- Class Ic, n (%)	0 (0)	
- Class III, n (%)	3 (19)	
Prior catheter ablation procedures, n (%)	0 (0%)	<0.001
Left atrial diameter (mm)	44 (40–46)	0.4
Left ventricular function, n (%)		
- Normal (ejection fraction 50–70%) - Mild dysfunction (40–49%) - Moderate dysfunction (30–39%) - Severe dysfunction (<30%)	14 (88) 1 (6) 1 (6) 0 (0)	0.3
**Procedural data**		
Total procedure time (min)	76 ± 31	0.095
Total fluoroscopy time (min)	14 ± 7	0.94
Three-dimensional mapping, n (%) High-density mapping, n (%)	16 (100) 6 (38)	0.1
**Total PFA applications per PV per patient**		0.12
**LSPV**	8 [8–8]	
**LIPV**	8 [8–8]	
**RSPV**	8 [8–8.25]	
**RIPV**	8 [8–8]	
**PFA catheter size**		
31 mm 35 mm	14 (85) 2 (15)	0.1
**Additional PFA ablation**	0 (0)	<0.001
**Additional RF ablation** CTI ablation	0 (0) 0 (0)	0.1
**All complications, n (%)**	0 (0)	0.1
Pericardial tamponade, n (%)	0 (0.0)	
Stroke/TIA	0 (0.0)	
Phrenic nerve palsy, n (%)	0 (0.0)	
Esophageal lesion, n (%)	0 (0.0)	
Coronary spasm, n (%)	0 (0.0)	
Minor groin complications, n (%)	0 (0.0)	

Values are mean ± standard deviation, median [first-third quartile] or n AAD, antiarrhythmic drug; AF, atrial fibrillation; BMI, body mass index. CHA_2_DS_2_-VASc score is a clinical estimation of the risk of stroke in patients with atrial fibrillation; scores range from 0 to 9, with higher scores indicating a greater risk of stroke = congestive heart failure, hypertension, age > 75 years, diabetes, previous stroke, transient ischemic attack, or thromboembolism, vascular disease, age 65–75 years and sex category. PAF, paroxysmal atrial fibrillation; LIPV, left inferior pulmonary vein; LSPV, left superior pulmonary vein; min, minutes; PFA, pulsed field ablation, PVI, pulmonary vein isolation; RSPV, right superior pulmonary vein; RIPV, right inferior pulmonary vein.

## Data Availability

All relevant data are within the manuscript.

## Abbreviations

AF	Atrial fibrillation
CA	Catheter ablation
LA	Left atrium/left atrial
LAPWI	Left atrial posterior wall isolation
LIPV	Left inferior pulmonary vein
LSPV	Left superior pulmonary vein
PersAF	Persistent atrial fibrillation
PFA	Pulsed Field Ablation
PV	Pulmonary vein
PVI	Pulmonary vein isolation
Pts	Patients
RIPV	Right inferior pulmonary vein
RSPV	Right superior pulmonary vein
